# Detecting the subtle shape differences in hemodynamic responses at the group level

**DOI:** 10.3389/fnins.2015.00375

**Published:** 2015-10-26

**Authors:** Gang Chen, Ziad S. Saad, Nancy E. Adleman, Ellen Leibenluft, Robert W. Cox

**Affiliations:** ^1^Scientific and Statistical Computing Core, National Institute of Mental Health, National Institutes of Health, Department of Health and Human ServicesBethesda, MD, USA; ^2^Department of Psychology, The Catholic University of AmericaWashington, DC, USA; ^3^Section on Bipolar Spectrum Disorders, Emotion and Development Branch, National Institute of Mental Health, National Institutes of Health, Department of Health and Human ServicesBethesda, MD, USA

**Keywords:** hemodynamic response, basis function, multivariate general linear model, linear mixed-effects model, FMRI group analysis, AFNI

## Abstract

The nature of the hemodynamic response (HDR) is still not fully understood due to the multifaceted processes involved. Aside from the overall amplitude, the response may vary across cognitive states, tasks, brain regions, and subjects with respect to characteristics such as rise and fall speed, peak duration, undershoot shape, and overall duration. Here we demonstrate that the fixed-shape (FSM) or adjusted-shape (ASM) methods may fail to detect some shape subtleties (e.g., speed of rise or recovery, or undershoot). In contrast, the estimated-shape method (ESM) through multiple basis functions can provide the opportunity to identify some subtle shape differences and achieve higher statistical power at both individual and group levels. Previously, some dimension reduction approaches focused on the peak magnitude, or made inferences based on the area under the curve (AUC) or interaction, which can lead to potential misidentifications. By adopting a generic framework of multivariate modeling (MVM), we showcase a hybrid approach that is validated by simulations and real data. With the whole HDR shape integrity maintained as input at the group level, the approach allows the investigator to substantiate these more nuanced effects through the unique HDR shape features. Unlike the few analyses that were limited to main effect, two- or three-way interactions, we extend the modeling approach to an inclusive platform that is more adaptable than the conventional GLM. With multiple effect estimates from ESM for each condition, linear mixed-effects (LME) modeling should be used at the group level when there is only one group of subjects without any other explanatory variables. Under other situations, an approximate approach through dimension reduction within the MVM framework can be adopted to achieve a practical equipoise among representation, false positive control, statistical power, and modeling flexibility. The associated program 3dMVM is publicly available as part of the AFNI suite.

## Introduction

When a region in the brain is activated, oxygen and glucose demands lead to blood vessel dilation, followed by increased blood to the tissue (neurons and astrocytes) under stress. The onset of a neuronal activity triggers a sequence of physiological events in the blood vessels of the surrounding area, typically characterized by the changes in cerebral blood flow as well as concentration fluctuations of deoxyhemoglobin and oxyhemoglobin. The blood oxygenation level dependent (BOLD) signal from the FMRI scanning mainly captures the concentration changes of deoxyhemoglobin; that is, the BOLD signal is a surrogate and signature of neuronal activations plus various sources of noise (e.g., physiological and random fluctuations). As an indirect measure of neuronal activity, the shape of the BOLD response may hold some crucial features about brain function. However, the cascade of events from neural activation to measurable MRI signal is complex and nonlinear under certain regimes (Friston et al., 1998b; Birn et al., 2001; Logothetis and Wandell, 2004; Logothetis, 2008; Magri et al., 2012): Even though the BOLD response is simply interpreted as changes in neuronal processing, the same neuronal activity may evoke different hemodynamic response (HDR) shape across trials, regions, conditions/tasks, subjects, and groups. For example, neurophysiological confounds such as neurovascular coupling or energy consumption changes could lead to different BOLD response features, potentially explaining the HDR variability in magnitude and shape across brain regions, cognitive conditions and populations (e.g., children with autism vs. controls, Reynell and Harris, 2013). Nevertheless, meaningful interpretation as well as detection power in FMRI data analysis may depend on the accurate modeling of the BOLD response both at the individual subject and group levels (e.g., Buxton et al., 2004; Handwerker et al., 2004; Stephen et al., 2007; Barbé et al., 2012; Badillo et al., 2013).

Under an experimentally-manipulated situation, the subject typically performs some tasks or is put under certain conditions in an event-related design, with each trial lasting for 2 s or less, and the HDR to each trial can be mathematically characterized by an impulse response function (IRF) that corresponds to a stimulus with a theoretically instantaneous duration and unit intensity. The voxel-wise EPI signal is then modeled through time series regression with explanatory variables (or regressors) of interest, each of which is constructed through the convolution between the stimulus timing and the IRF. In a block design, each task or condition has a duration of more than two seconds. As each block can be approximately considered as a sequence of events with an interval of scanning repetition time (TR), the theoretical HDR is usually hypothesized as the integral or linear summation of the consecutive IRFs, or the convolution of IRF over the stimulus duration.

We typically adopt some formative mathematical functions (usually called HDR functions or HRFs) to approximate the HDR based on the experimental data with the assumption of linearity and time-invariance (or stationarity) (Marrelec et al., 2003), and consider three common approaches to modeling the average HDR across trials. The first one presumes a fixed shape IRF (e.g., gamma variate or wave form in AFNI, Cohen, 1997; canonical IRF in SPM, FSL, and NIPY, Friston et al., 1998a). With this model-based or fixed-shape method (FSM), the regression coefficient or β associated with each condition in the individual subject analysis reflects the major HDR magnitude (e.g., percent signal change). The second approach makes no assumption about the IRF's shape and estimates it with a set of basis functions. The number of basis functions varies depending on the kernel set and the duration over which the response is being modeled. A common approach to this estimated-shape method (ESM) consists of using a set of equally-spaced TENT (piecewise linear) functions or linear splines, and each of the resulting regression coefficient represents an estimate of the response amplitude at some time after stimulus onset. Regardless of the kernel set, however, ESM generates the same number of regressors as the number of basis functions (e.g., *m*) per condition or task, resulting in *m* regression coefficients which need to be considered simultaneously at the group level. In addition to the aforementioned TENT basis set, options for ESM at the voxel level include cubic splines, Legendre polynomials, sines, or user-defined functions in AFNI, and finite impulse function (FIR) in SPM, FSL, and NIPY, inverse logit (Lindquist et al., 2009), and high-order B-splines (Degras and Lindquist, 2014). In addition, the python package PyHRF offers an ESM at the parcel level through the joint detection-estimation framework (Vincent et al., 2014). It is of note that one significant advantage of adopting basis functions such as TENT or cubic splines is the flexibility of creating regressors through piecewise interpolation when the stimulus onset times are not aligned with the TR grids (e.g., the acquisition time is shorter than TR if one wants to present “silent trials” as a control condition to speech or other auditory stimulus). The third approach lies between the two extremes of FSM and ESM, and uses a set of two or three basis functions (Friston et al., 1998b). In this adjusted-shape method (ASM), the first basis (canonical IRF) captures the major HDR shape, and the second basis, the time derivative of the canonical IRF, provides some flexibility in modeling the delay or time-to-peak, while the third basis, dispersion curve (derivative relative to the dispersion parameter in the canonical IRF), allows the peak duration to vary.

With one parameter per condition, FSM is the most efficient^1^ and statistically powerful among the three, if the presumed shape is reasonably close to the ground truth, and the group analysis strategies have been developed to reasonable maturity: The β values at the individual level are typically brought to the group level using the Student's *t*-test, permutation tests (Nichols and Holmes, 2002; Dehaene-Lambertz et al., 2006; Mériaux et al., 2006; Winkler et al., 2014), AN(C)OVA, general linear model (GLM) (Poline and Brett, 2012), multivariate modeling (MVM) (Chen et al., 2014), linear mixed-effects (LME) method (Bernal-Rusiel et al., 2013; Chen et al., 2013), or mixed-effect multilevel analysis (Worsley et al., 2002; Woolrich et al., 2004; Chen et al., 2012), with the assumption that each effect estimate is equally reliable across all subjects. However, deviations of the HDR from the presumed shape would result in biased estimates of the amplitude, in addition to failing to capture differences in shape such as during the undershoot or recovery phase. ESM is the most flexible among the three methods in terms of providing a more accurate characterization of the BOLD response and can achieve higher activation detection power in individuals. In addition, the estimated HDR curve with a unique signature shape offers much stronger support for the existence of activation than a single scaling factor or β value with FSM or ASM. Compared with FSM, ASM also results in a less biased response amplitude for the principal kernel, and can account for more variance compared to FSM; however, the common practice of using only the principal kernel's coefficient at the group level will not allow the detection of shape changes between conditions and or groups when those exist.

Difficulties with using ESM (and to a lesser degree ASM) include the need for a larger number of kernel coefficients that need to be estimated. They requires *m* times more regressors than FSM in the individual subject analysis, which translates to more data points and scanning time to reach similar statistical power in individuals. Secondly, the risk of over-fitting exists when some confounding effects such as head motion and physiological noise are stimulus-locked and not fully accounted for. Lastly, the most challenging step lies at the group level: How to simultaneously handle those *m* effect estimates? And how to summarize and interpret the results? To avoid the complexity involved in the multiple effect estimates from ESM or ASM, the popular approach at the group level is dimensional reduction, condensing the shape information over the multiple values into one number. For ESM, one method is to sum over all or a subset of effect estimates (e.g., ignoring a few time points at the beginning and the end) to obtain the area under the curve (AUC) (e.g., Beauchamp et al., 2003; Greene et al., 2007; McGregor et al., 2013). As the BOLD response curve can be characterized by parameters such as amplitude (or height), delay (or time-to-peak), duration (or HWFM), another dimensional reduction proposal is to perform the group analysis on such a derived parameter from the estimated HDR (Lindquist et al., 2009; Degras and Lindquist, 2014). With two or three effect estimates per condition from ASM at the group level, the popular approach focuses on the β value of the canonical HDR while ignoring the parameters for the shape adjustments (i.e., the function of these other parameters is to absorb minor shape fluctuations that would otherwise be modeled as “noise”). One alternative is to estimate the HDR height using the Euclidean or *L*^2^-norm distance (L2D) of the two or three effect estimates (Calhoun et al., 2004; Lindquist et al., 2009; Steffener et al., 2010). Essentially, these dimensional reduction methods transform the effect estimates in an *k*-dimensional space ℝ^*k*^ to one-dimensional ℝ^1^. As information loss is unavoidable in the process, statistical power in activation identification would suffer. This raises the question of whether a more preferable approach to significance testing might better exploit the information in the HDR shape at the group level.

### A motivational example

To demonstrate and compare various modeling approaches at the group level, we adopt the same experimental data used in our previous paper (Chen et al., 2014), with a typical group design that accounts for a confounding effect: varying age across subjects. Briefly, the experiment involved one between-subjects factor, group (two levels: 21 children and 29 adults) and one within-subject factor (two levels: congruent and incongruent conditions). Stimuli were large letters (either “H” or “S”) composed of smaller letters (“H” or “S”). For half of the stimuli, the large letter and the component letters were congruent (e.g., “H” composed of “H”s) and for half they were incongruent (e.g., “H” composed of “S”s). Parameters for the whole brain BOLD data on a 3.0 T scanner were: voxel size of 3.75 × 3.75 × 5.0 mm^3^, 24 contiguously interleaved axial slices, and TR of 1250 ms (TE = 25 ms, FOV = 240 mm, flip angle = 35°). Six runs of EPI data were acquired from each subject, and each run lasted for 380 s with 304 data points. The task followed an event-related design with 96 trials in each run, with three runs of congruent stimuli interleaved with three runs of incongruent stimuli (order counterbalanced across subjects). Subjects used a two button box to identify the large letter during global runs and the component letter during local runs. Each trial lasted 2500 ms: the stimulus was presented for 200 ms, followed by a fixation point for 2300 ms. Inter-trial intervals were jittered with a varying number of TRs, allowing for a trial-by-trial analysis of how the subject's BOLD response varied with changes in reaction time (RT). The experiment protocol was approved by the Combined Neuroscience Institutional Review Board at the NIMH, and the National Clinical Trials Identifier is NCT00006177.

The EPI time series went through the following preprocessing steps: slice timing and head motion corrections, spatial alignment to a Talairach template (TT_N27) at a voxel size of 3.5 × 3.5 × 3.5 mm^3^, smoothing with an isotropic FWHM of 6 mm, and scaling each voxel time series by its mean value. The scaling step during preprocessing enables one to interpret each regression coefficient of interest as an approximate estimate of percent signal change relative to the temporal mean. The six runs of data were concatenated for the individual regression analysis with the discontinuities across runs properly handled (Chen et al., 2012). To capture the subtle HDR shape under a condition, two modeling approaches were adopted, ESM and ASM, for model comparison. With ESM, each trial was modeled with 10 tent basis functions, each of which spanned one TR (or 1.25 s). The subject's RT at each trial was incorporated as a per-trial modulation variable. In other words, two effects per condition were estimated in the time series regression at the individual level: one revealed the response curve associated with the average RT while the other showed the marginal effect of RT (response amplitude change when RT increases by 1 s) at each time point subsequent to the stimulus. In addition, the following confounding effects were included in the model for each subject, for each run: third-order Legendre polynomials accounting for slow drifts, incorrect trials (misses), censored time points with extreme head motion, and the six head motion parameters. The modeling strategy remained the same with ASM except that the three SPM basis functions (canonical IRF plus time and dispersion derivatives) were employed to model the BOLD responses instead of the 10 tents.

At the group level, it is the BOLD effects associated with the average RT that are of interest here. In addition to the estimated HDR profiles, three other explanatory variables considered are: a) between-subjects factor, Group (two levels: children and adults), b) within-subject factors, Condition (two levels: congruent and incongruent), and c) quantitative covariate, age. The focus is on the interaction of HDR between Group and Condition: Do the two groups differ in the HDR profile contrast between the two conditions?

### Preview

This paper is a sequel to our previous exploration (Chen et al., 2014) of the multivariate modeling (MVM) approach for FMRI group analysis. The layout is as follows. First, we explore and review various hypothesis testing strategies at the group level when the HDR is estimated through multiple basis functions. Second, simulation data were generated to reveal how each methodology performs in terms of controllability for false positives and false negatives, and the performance of these methods was assessed when they were applied to the experimental dataset at both individual and group levels. Finally, we compare all the modeling methodologies for ASM and ESM as well as with and without dimension reduction. The modeling strategies and testing methods discussed here are all performed at the voxel level. Multiple testing correction can be applied in the conventional fashion by controlling the false positive rate (Benjamini and Hochberg, 1995) or the family-wise error through Monte Carlo simulations (3dClustSim in AFNI, Forman et al., 1995) or random field theory (Worsley et al., 1992).

Our major contribution here is to demonstrate the importance of accounting for shape differences and to offer testing approaches at the group level within an MVM platform with the modeling flexibility that would not be available under the conventional GLM. Through our demonstration we propose that ESM should be adopted whenever appropriate or possible to identify the nuanced differences in HDR shape that would be difficult or unlikely to be revealed through FSM or ASM. Furthermore, we recommend that the investigator report the effect estimates such as the HDR curves to substantiate the results in addition to the statistical significance. The modeling framework and functionality are available in the program 3dMVM for public use in the AFNI suite (Cox, 1996).

Throughout this article, regular italic letters (e.g., α) stand for scalars, boldfaced italic letters in lower (***a***) and upper (***X***) cases for column vectors and matrices respectively. The word *multivariate* is used here in the sense of treating the effect estimates from the same subject or from the levels of a within-subject factor as the instantiations of simultaneous response (or outcome) variables (e.g., the effect estimates for the HDR). This usage differs from the popular connotation in the FMRI field when the spatial structure (multiple voxels) is modeled as the simultaneous response variables, including such methods as multivariate pattern analysis (Haxby, 2012), independent component analysis, and machine learning methods such as support vector machines. Major acronyms used in the paper are listed in Appendix A.

## Methods

As shown in Chen et al. (2014), we formulate the group analysis under a multivariate GLM or MVM platform that is expressed from a subject-wise perspective, $\beta_{i}^{T}=x_{i}^{T}A+\delta_{i}^{T}$, or through the variable-wise pivot, ***b***_*j*_ = ***Xa***_*j*_ + ***d***_*j*_, or in the following concise form,

(1) $$B_{n\times m}=X_{n\times q}\;A_{q\times m}+D_{n\times m}.$$

The *n* rows of the response matrix $B={\left(\beta_{ij}\right)}_{n\times m}={\left(\beta_{1}^{T},\beta_{2}^{T},...,\beta_{n}^{T}\right)}^{T}=(b_{1},b_{2},...,b_{m})$ represent the data from the *n* subjects while the *m* columns correspond to the levels of within-subject factor(s). For example, the effect estimates from the multiple basis functions under ESM or ASM can be considered the response values associated with the levels of a within-subject or repeated-measures factor (termed Component hereafter). When multiple within-subject factors occur, all their level combinations for each subject are *flattened* from a multi-dimensional space onto a one-dimensional row of ***B***. It is noteworthy that the within-subject factors are expressed as columns in ***B*** on the left-hand side of the model (1), and only between-subjects variables such as subjects-grouping factors (e.g., sex, genotypes), subject-specific measures (e.g., age, IQ) and their interactions are treated as *q* explanatory variables on the right-hand side. The same linear system is assumed for all the *m* response variables, which share the same design matrix $X=(x_{ih})={\left(x_{1},x_{2},...,x_{n}\right)}^{T}$. Without loss of generality, ***X*** is assumed to have full column-rank *q*. Each column of the regression coefficient matrix ***A*** = (α_*hj*_) corresponds to a response variable, and each row is associated with an explanatory variable. Lastly, the error matrix $D={\left(\delta_{ij}\right)}_{n\times m}={\left(\delta_{1},\delta_{2},...,\delta_{n}\right)}^{T}=(d_{1},d_{2},...,d_{m})$ is assumed *nm*-dimensional Gaussian: *vec*(***D***) ~*N*(***0**, **I***_*n*_ ⊗ **Σ**), where *vec* and ⊗ are column stacking and direct (or Kronecker) product operators respectively. As in univariate modeling (UVM), the assumptions for model (1) are linearity, Gaussianity and homogeneity of variance-covariance structure (same **Σ** across all the between-subjects effects). When only one group of subjects is involved (*q* = 1), the parameter matrix ***A*** becomes a row vector (α_1_, α_2_, …, α_*m*_) that is associated with the *m* levels of a within-subject factor.

As demonstrated in Chen et al. (2014), MVM has a few advantages over its univariate counterpart. When the data are essentially multidimensional like the multiple effect estimates from ESM or ASM, MVM has a crucial role in formulating hypothesis testing. In addition, it characterizes and quantifies the intercorrelations among the variables based on the data rather than a presumed variance-covariance structure as in UVM. Furthermore, MVM in general provides a better control for false positives than UVM. Lastly, the conventional univariate testing (UVT) under GLM can be easily performed under the MVM framework with a few extra advantages. Here we discuss one aspect by which the group analysis of neuroimaging data will benefit from the MVM facility when the HDR profile is estimated from multiple basis functions instead of being presumed to have a fixed shape. Then in the section Simulations and Real Experiment Results, we elaborate and compare a few testing alternatives in terms of power and false positives, using simulations and in terms of performance with real data.

### Different testing strategies

Here we exemplify two simple and prototypical cases with the HDR profile modeled by *m* basis functions at the individual subject level: a) one group of subjects with the associated effects at the group level expressed as α_1_, α_2_, …, α_*m*_ under (1), and b) either two groups or two conditions and the two sets of effect estimates for HDR are α_1*j*_ and α_2*j*_ respectively, *j* = 1, 2, …, *m*. To simplify geometric representations, we assume equal number of subjects across groups in the case of group comparison, but the assumption is not required from the modeling perspective. The various modeling strategies discussed below for these two cases can be easily extended to situations with more explanatory variables, including factors and quantitative covariates.

#### Multivariate testing (MVT)

As the analogs of one- and two-sample or paired *t*-tests under UVT, the two prototypes can be expressed with the following null hypotheses,

(2a) $$\;H_{01}^{MVT}:\alpha_{1}=0,\alpha_{2}=0,...,\alpha_{m}=0,$$

(2b) $$H_{02}^{MVT}:\alpha_{11}=\alpha_{21},\alpha_{12}=\alpha_{22},...,\alpha_{1m}=\alpha_{2m}.$$

In other words, the *m* regression coefficients associated with the *m* basis functions from each subject are brought to the group level and treated as the instantiated values of *m* simultaneous variables. When the effect estimates associated with the basis functions of ESM or ASM are treated as the values of *m* simultaneous response variables, the hypothesis (2a) or (2b) can be analyzed through MVT under the model (1). Geometrically, the data for $H_{01}^{MVT}$ represent the group centroid (α_1_, α_2_, …, α_*m*_) in the *m*-dimensional real coordinate space ℝ^*m*^ (Table 1), and the associated one-sample Hotelling *T*^2^-test is performed to reveal whether the group centroid lies in the rejection region (outside of an *m*-dimensional ellipse centering around the origin in the case of $H_{01}^{MVT}$). Similarly, the data for $H_{02}^{MVT}$ are expressed as two group centroids, (α_11_, α_12_, …, α_1*m*_) and (α_21_, α_22_, …, α_2*m*_), and the corresponding two-sample Hotelling *T*^2^-test is conducted to see if the hypothesis (2b) about the two centroids can be rejected. The hypothesis (2b) can be easily generalized to the situation with more than two groups of subjects (e.g., three genotypes) as well as more than one subject-grouping variable (e.g., sex, genotypes, and handedness) through the formulation of general linear testing (Chen et al., 2014). One noteworthy feature of MVT is that it allows those simultaneous effects to have different scales or units, unlike the traditional AN(C)OVA or univariate GLM in which all the levels of a factor are usually of the same dimension.

**Table 1:** **Schematic comparisons among various testing methods**.

**One-sample**				
**Method^*a*^**	**MVT/LME**	**AUC**	**L2D**	**EXC (XUV and XMV)**
*H*_0_	α_1_ = … = α_*m*_ = 0	α_1_ + … + α_*m*_ = 0	${\left(\alpha_{1}^{2}+\ldots +\alpha_{m}^{2}\right)}^{1∕2}=0$	α_1_ = … = α_*m*_
Dimensions in ℝ^*m*^	0	*m*−1	*m*−1	1
DFs for *F*-statistic^*b*^	*m, n*−*m*−*q*+1	1, *n*−*q*	1, *n*−*q*	*m*−1, (*m*−1)(*n*−*q*)
Geometric representation^*c*^ of *H*_0_ and *H*_1_ (*m* = 2)	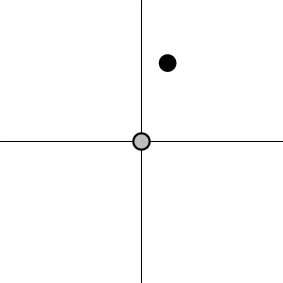	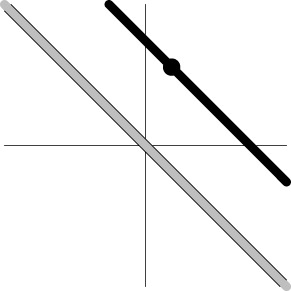	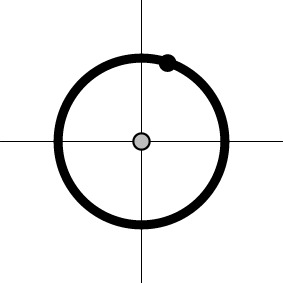	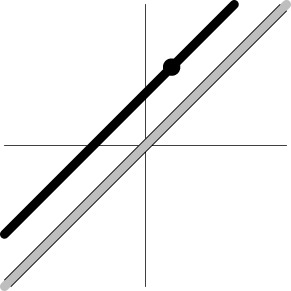
Geometric representation^*d*^ of HDR when detection failure occurs due to improper *H*_0_ formulation	no	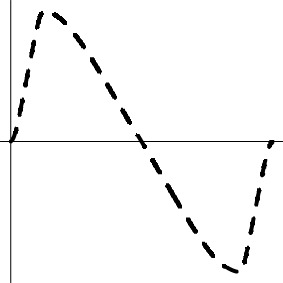	no	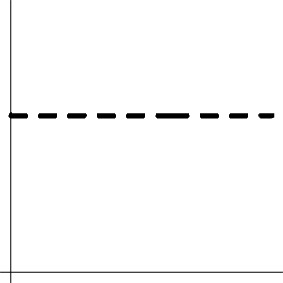
**Two-sample or paired**				
**Method**	**MVT**	**AUC**	**L2D**	**EXC (XUV and XMV)**
*H*_0_	α_11_ = α_21_, …, α_1*m*_ = α_2*m*_	$\overset{m}{\underset{j=1}{\sum }}\alpha_{1j}=\overset{m}{\underset{j=1}{\sum }}\alpha_{2j}$	${\left(\overset{m}{\underset{j=1}{\sum }}\alpha_{1j}^{2}\right)}^{1∕2}={\left(\overset{m}{\underset{j=1}{\sum }}\alpha_{2j}^{2}\right)}^{1∕2}$	α_11_ − α_21_ = … = α_1*m*_ − α_2*m*_
Dimensions in ℝ^*m*^	0	*m*−1	*m*−1	1
DFs for *F*-statistic	*m, n*−*m*−*q*+1	1, *n*−*q*	1, *n*−*q*	*m*−1, (*m*−1)(*n*−*q*)
Geometric representation^*e*^ of *H*_0_ and *H*_1_	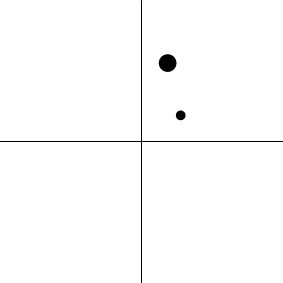	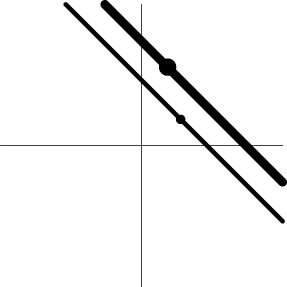	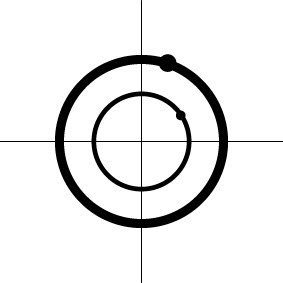	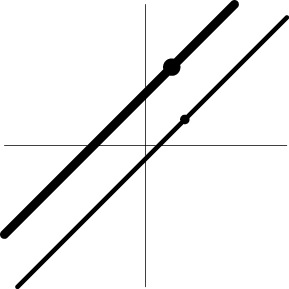
Geometric representation^*f*^ of HDR when detection failure occurs due to improper *H*_0_ formulation	no	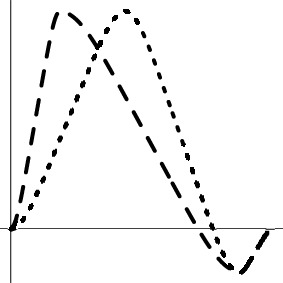	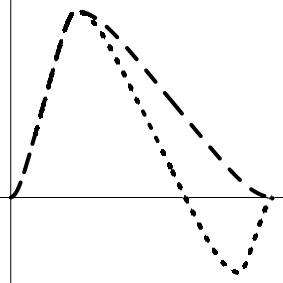	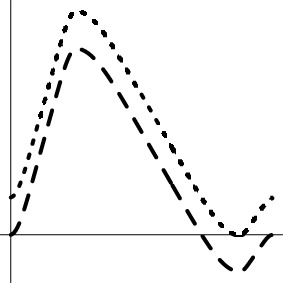

a *The table is meant to show the dimensions of each null hypothesis and an instantiation in the rejection domain while the whole rejection domain is not represented here. For example, the reject region of one-sample Hotelling T^2^-test for MVT (2a) is outside of an m-dimensional ellipse*.

b *An interesting fact is that the numerator degrees of freedom for the F-statistic under MVT and UVT are the dimensions of the complementary space to the associated null hypothesis H_0_, or the dimensions of the alternative hypothesis H_1_*.

c *The two axes represent the two weights associated with the two basis functions. The whole rejection regions are not shown here, and the shaded (gray) and solid (black) areas correspond respectively to the null hypothesis H_0_ space and an instantiation (and its dimension) in the alternative hypothesis H_1_ space. Detection failure occurs when the group centroid falls on the diagonal line other than the origin under AUC and EXC*.

d *The horizontal and vertical axes represent time and the amplitude of HDR curve (dashed line)*.

e *The two axes represent the two weights associated with the two basis functions. The whole rejection regions are not shown here, and the shaded and sold areas correspond respectively to the null hypothesis H_0_ space and an instantiation (and its dimension) in the alternative hypothesis H_1_ space. The two types of line thickness (or dot size) differentiate the two groups (or conditions)*.

f *The horizontal and vertical axes represent time and the amplitude of HDR curves. The two line types, dashed and dotted, differentiate the two groups or conditions*.

#### Linear mixed-effects modeling (LME)

As demonstrated in Chen et al. (2013), linear mixed-effects modeling (LME) can be adopted for group analysis when the HDR is estimated through multiple basis functions. Specifically, the *m* regression coefficients from each subject associated with the *m* basis functions are modeled as values corresponding to *m* levels of a within-subject factor under the LME framework. When no other explanatory variables are present in the model, the LME methodology can be formulated by (2a) with an intercept of 0. That is, the *m* effects are coded by *m* indicator variables instead of any conventional contrast coding. Suppose that the *m* effect estimates associated with the *m* basis functions from the *i*th subject are β_*i*1_, β_*i*2_, …, β_*im*_, the LME model can be specified as,

$$\beta_{ij}=\alpha_{j}x_{ij}+\delta_{i}+\epsilon_{ij},i=1,2,...,n,j=1,2,...,m.$$

where the random effect δ_*i*_ characterizes the deviation or shift of the *i*th subject's HDR from the overall group HDR, the residual term ϵ_*ij*_ indicates the deviation of each effect estimate β_*ij*_ from the *i*th subject's HDR, and the indicator variables *x*_*ij*_ take the cell mean coding,

$${\text{x}}_{\text{ij}}\text{=}\{\begin{array}{ll} \text{1,} & \text{if }i\text{th subject is at }j\text{th level},\\ 0, & \text{otherwise.} \end{array}$$

so that the parameters α_*j*_, *j* = 1, 2, …, *m* capture the overall group HDR. The significance of the overall HDR at the group level can be tested through LME on the same hypothesis as (2a),

(3) $$H_{0}^{LME}:\alpha_{1}=0,\alpha_{2}=0,...,\alpha_{m}=0.$$

It is of note that the LME approach does not work when other explanatory variables (multiple groups, conditions, or quantitative covariates) are involved because (2a) or (2b) cannot be formulated due to the parameterization constraint through dummy coding. For instance, when there are two groups involved, the typical contrast coding for the two groups renders one dummy variable (e.g., the contrast of one group vs. the other when effect coding is adopted); however, such a coding strategy relies on the existence of an intercept in the model. If the two groups are coded by two indicator variables, the model matrix would become overparameterized.

#### Area-under-the-curve (AUC)

The multiple estimates associated with the multiple basis functions can be reduced to a single value, which is the area under the curve of the estimated response function. The AUC hypotheses for the two prototypes (2a) and (2b) become

(4a) $$H_{01}^{AUC}:\sum_{j=1}^{m}\alpha_{j}\;=\;0,$$

(4b) $$H_{02}^{AUC}:\sum_{j=1}^{m}\alpha_{1j}\;=\;\sum_{j=1}^{m}\alpha_{2j}.$$

That is, the sum of the *m* coefficients (or area under the HDR curve) is used to summarize the overall response amplitude per subject in one- or two-sample *t*-test at the group level. The AUC hypotheses (4a) and (4b) are essentially a zero-way interaction (or intercept) and a one-way interaction (or the main effect of Group or Condition) respectively and can be performed under the AN(C)OVA, GLM, or MVM framework. Their geometrical interpretations are as follows (cf. Table 1). The data for $H_{01}^{AUC}$ lie on an ℝ^*m*−1^ isosurface (or hyperplane) α_1_ + … + α_*m*_ = *c*, and the associated test for AUC (4a) is executed on the distance between the data isosurface and the null isosurface α_1_ + … + α_*m*_ = 0. As the correct null hypothesis for MVT (2a) is only a subset of AUC (4a), the rejection domain of AUC (4a) is only a subset of the rejection domain for MVT (2a), leading to a misrepresentation in (4a) and a detection failure when a data point lies on α_1_ + … + α_*m*_ = 0 but not at the origin (i.e., the HDR curve has roughly equal area below and above the *x*-axis, e.g., a large undershoot). Similarly for $H_{02}^{AUC}$.

#### Euclidean distance (L2D)

As an alternate dimension reduction approach, the null hypotheses associated with the Euclidean or *L*^2^ distance (L2D) for ESM can be formulated respectively as

(5a) $$H_{01}^{L2D}:{\left(\sum_{j=1}^{m}\alpha_{j}^{2}\right)}^{1/2}=0,$$

(5b) $$H_{02}^{L2D}:{\left(\sum_{j=1}^{m}\alpha_{1j}^{2}\right)}^{1/2}={\left(\sum_{j=1}^{m}\alpha_{2j}^{2}\right)}^{1/2}.$$

In other words, one captures the overall magnitude for each subject using the *L*^2^-distance of the *m* regression coefficients from no response, and then performs one- or two-sample *t*-test on the distances.

For ASM, the null hypotheses with the focus on the canonical basis are

(6a) $$H_{0}^{CAN}:\alpha_{1}=0,$$

(6b) $$H_{0}^{CAN}:\alpha_{11}=\alpha_{21}.$$

And the null hypotheses for L2D (Calhoun et al., 2004; Steffener et al., 2010) are tested with the first two bases,

(7a) $$H_{0}^{L2D}:sgn(\alpha_{1}){\left(\alpha_{1}^{2}+\alpha_{2}^{2}\right)}^{1/2}=0,$$

(7b) $$H_{0}^{L2D}:sgn(\alpha_{11}){\left(\alpha_{11}^{2}+\alpha_{12}^{2}\right)}^{1/2}=sgn(\alpha_{21}){\left(\alpha_{21}^{2}+\alpha_{22}^{2}\right)}^{1/2}$$

or with all the three bases,

(8a) $$H_{0}^{L2D}:sgn(\alpha_{1}){\left(\alpha_{1}^{2}+\alpha_{2}^{2}+\alpha_{3}^{2}\right)}^{1/2}=0,$$

(8b) $$\begin{array}{l} H_{0}^{L2D}:sgn(\alpha_{11}){\left(\alpha_{11}^{2}+\alpha_{12}^{2}+\alpha_{13}^{2}\right)}^{{}^{1/2}}=sgn(\alpha_{21})\\ \;{\left(\alpha_{21}^{2}+\alpha_{22}^{2}+\alpha_{23}^{2}\right)}^{{}^{1/2}}, \end{array}$$

where *sgn* is the sign function. That is, the L2D for ASM is similar to the L2D for ESM, but using the two or three weights associated with the two or three basis functions in ASM and assigning the sign of the canonical response to the resultant *L*^2^-distance.

Their geometrical interpretations are as follows (Table 1). The data for $H_{01}^{L2D}$ lie on an ℝ^*m*−1^ iso-sphere, and the associated test for (5a) is executed on the radius of the ℝ^*m*−1^ iso-sphere, leading to no geometrical distortion (but not necessarily true statistically). On the other hand, the data for $H_{02}^{L2D}$ are on two ℝ^*m*−1^ iso-sphere surfaces, and the associated test for (5b) acts on the radius difference between the two ℝ^*m*−1^ iso-spheres, resulting a detection failure when the two HDR curves have roughly the same radius.

#### Effect-by-component interaction (EXC: XUV and XMV)

By treating the *m* effect estimates from ESM as *m* levels of a within-subject factor Component, one can test the hypothesis for the effect-by-component interaction (EXC); that is, the *m* regression coefficients associated the *m* basis functions are taken to the group level without any condensation:

(9a) $$H_{01}^{EXC}:\alpha_{1}=\alpha_{2}=...=\alpha_{m},$$

(9b) $$H_{02}^{EXC}:\alpha_{11}-\alpha_{21}=\alpha_{12}-\alpha_{22}=...=\alpha_{1m}-\alpha_{2m}.$$

As discussed in Chen et al. (2014), EXC (9) can be tested through two methods, one univariate testing for the interaction (XUV), and one multivariate testing for the interaction (XMV). More specifically, with XUV one tests the equality among the *m* components in (9) by treating them as the *m* levels of a within-subject factor in an AN(C)OVA or univariate GLM platform. In contrast, the equality among the *m* components in (9) is tested in XMV as *m* simultaneous variables in an MAN(C)OVA or multivariate GLM (Appendix B).

The geometrical interpretations of the hypotheses are the following (Table 1). EXC (9a) tests the main effect (or first-way interaction) of Component, representing a straight line in ℝ^*m*^. The associated test for (9a) is executed on the distance between the data line and the null line (a diagonal line through the origin). As the correct null hypothesis (2a) is only a subset of $H_{01}^{EXC}$, its rejection domain is only a subset of the rejection domain for MVT (2a), leading to a misrepresentation in (9a) and a detection failure when the group centroid lies on the null line but not at the origin (i.e., the HDR curve is roughly a flat line). Similarly, EXC (9b) as a two-way interaction between Group/Condition and Component is represented by two lines, and the corresponding test acts on the distance between the two lines: are the HDR profiles parallel with each other between the two groups or conditions? As the correct null hypothesis (2b) is only a subset of EXC (9b), the rejection domain of EXC (9b) is only a subset of MVT (2b), resulting in a misrepresentation in (9b) and a detection failure when the two HDR curves are roughly parallel with each other (Table 1).

## Simulations and real experiment results

Among all the testing strategies, LME and MVT are the most precise (points in Table 1). Among all the dimensional reduction methods, the two EXC methods, XUV and XMV, are of the closest approximation to the null hypothesis (lines), while AUC and L2D are the least accurate (ℝ^*m*−1^ planes and sphere surfaces respectively). We need to address the question of whether the geometric accuracy order translates to statistical power through simulations and to performance when the methods are applied to real data.

### Simulations of group analysis with different testing methods

**Figure 1:**
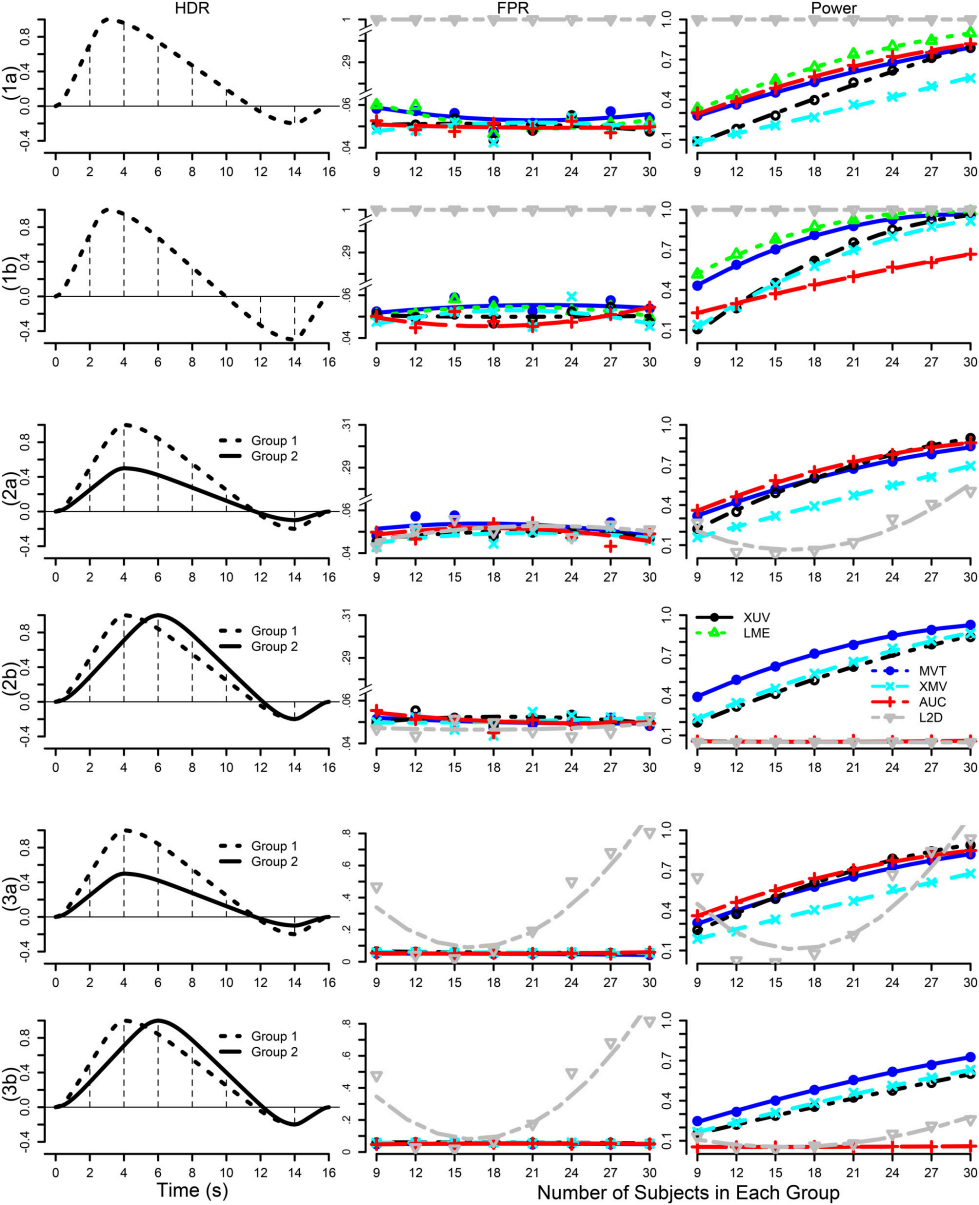
**Simulation parameters and results**. The six rows correspond to the scenarios in which the presumed HDRs (first column) with a poststimulus undershoot were generated by the convolution program waver in AFNI, and sampled at TR = 2 s (shown with vertical dotted lines): (1) one group with a small (1a, σ = 1.8) and a moderate (1b, σ = 1.8) undershoot, (2) two homoscedastic groups with the same HDR shape but different amplitudes (2a, σ = 0.5) and with same peak amplitude but a difference of two seconds in peak location (2b, σ = 0.3), (3) two heteroscedastic groups with the same HDR shape but different amplitudes (3a, σ = 0.3) and with same peak amplitude but a difference of two seconds in peak location (3b, σ = 0.3). FPR and power are shown in the second and third columns with a varying number of subjects in each group at a temporal correlation coefficient ρ of 0.3 under six testing approaches: XUV, LME, MVT, XMV, AUC, and L2D. The curves for FPR and power were fitted to the simulation results (plotting symbols) through LOESS smoothing with second order local polynomials.

As the spatial extent of FMRI data analysis is independently controlled through false positive rate or family-wise error, the simulations here were performed at a voxel to examine and compare the false positives and power performance among the testing methods. Simulated data were generated with the following parameters, imitating a typical FMRI group analysis with six scenarios (top row in Figure 1): a) one group of subjects with a small undershoot at the end of HDR curve; b) one group of subjects with a moderate undershoot at the end; c) two homoscedastic groups (same variance between groups) with equal number of subjects in each with a similar HDR profile but a factor of 2 difference in amplitude; d) two homoscedastic groups with equal number of subjects in each with HDR having the same amplitude but with a 2 s difference in peak location; e) two heteroscedastic groups (different variance between groups) with equal number of subjects in each with a similar HDR profile but a factor of 2 difference in amplitude; and f) two heteroscedastic groups with equal number of subjects in each with HDR having the same amplitude but with a 2 s difference in peak location. The HDRs are presumably estimated through 7 basis functions (e.g., TENT in AFNI) at the individual level, and the associated 7 effect components {β_*i*_, *i* = 1, 2, …, 7} at the TR grids are assumed to follow a multivariate Gaussian distribution with a first order autoregressive AR(1) structure for their variance-covariance matrix

$$\Sigma =\sigma^{2}\left[\begin{array}{c} \begin{array}{ccccc} 1 & \rho & \rho^{2} & ... & \rho^{6}\\ \rho & 1 & \rho & ... & \rho^{5}\\ \vdots & \vdots & \vdots & \vdots & \vdots \\ \rho^{6} & \rho^{5} & \rho^{4} & ... & 1 \end{array} \end{array}\right].$$

The choice of a simple Σ structure here is to allow manageable number of simulations while in the same time providing a reasonable structure similar to the one adopted for the Gaussian prior in Marrelec et al. (2003) that guarantees the HDR smoothness. To explore the impact of sample size, the number of subjects in each group was simulated at *n* = 9, 12, 15, 18, 21, 24, 27, 30 with ρ = 0.3 for each of the six scenarios. The standard error σ varied (shown in Figure 1) across the scenarios to obtain comparable power for each *n*. 5000 datasets were simulated, each of which was analyzed through 3dMVM with two explanatory variables, Group (between-subjects factor with 2 levels) and Component (within-subject factor with 7 levels that are associated with the 7 basis functions). False positive rate (FPR) and power were assessed by counting the datasets with their respective *F*- or *t*-statistic surpassing the threshold corresponding to the nominal significance level of 0.05. Similarly, one- or two-sample *t*-test was performed on the AUC and L2D values respectively.

Among the six scenarios, all the testing methods showed proper control of FPR except for L2D with one group of subjects. L2D exhibits high power but at the cost of poor FPR control. This is in part due to the reduction of effect estimates to a positive value regardless the signs of the individual components in ESM. It is possible to reduce this problem in ASM when the sign of the principal kernel is assigned to the resulting L2D measure as shown in (7) and (8). Also, L2D achieved the lowest power with two groups of subjects. AUC simply sums over all the components, significantly misrepresenting the effects when the undershoot becomes moderate. This is reflected in the results where reasonable power is achieved when the undershoot is small and lower power is obtained when the undershoot is moderate. With two groups, AUC performed well in power when the two groups had the same HDR shape, but behaved as poorly as L2D when the two groups had different HDR shapes. As expected, AUC is only sensitive to peak amplitude differences, but is insensitive to shape subtleties. Except for L2D and AUC, the other methods tend to converge in power when the sample size is large enough (e.g., 30 or more). With one group, LME outperformed all other candidates. XUV had a balanced performance on power among all the scenarios, constantly surpassing XMV. Lastly, MVT was slightly more powerful than XUV with two groups when their HDRs were of the same shape with a large number of subjects (e.g., 20 or more per group).

In summary, our simulations show that LME is preferred when there is only one group of subjects with no other explanatory variables present. Under other circumstances, XUV is the preferred choice, especially with the typical sample size of most studies, while MVT, AUC, and XMV may provide some auxiliary detection power.

### Results with experimental data

**Figure 2:**
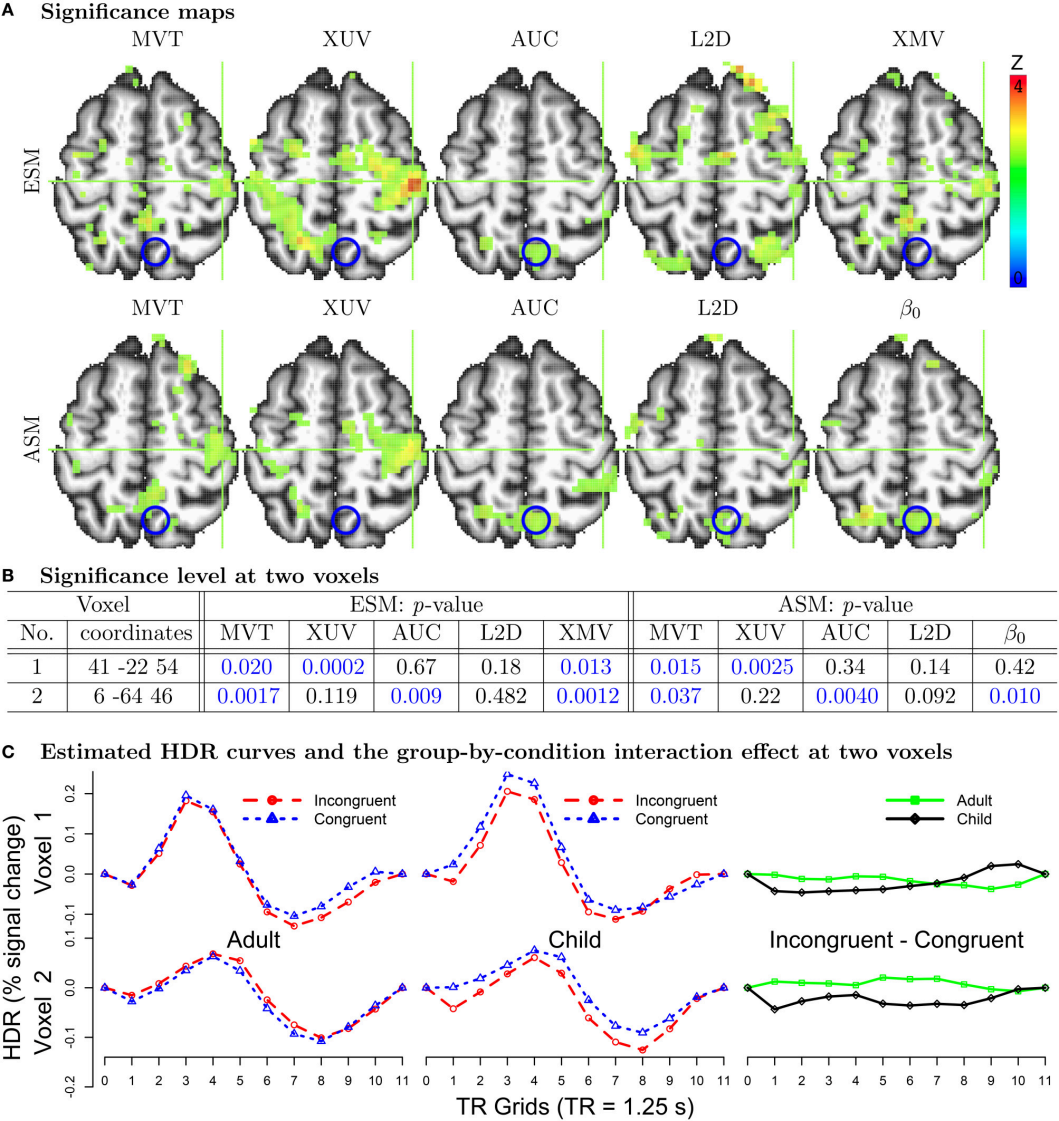
**Analysis results of experimental data**. **(A)** Five tests for ESM and ASM are illustrated at an axial slice (*Z* = 54 mm) at *p* = 0.05 level with the radiological convention (left is right). To demonstrate the subtle differences among the methods, the raw results are shown here without multiple testing correction applied. When family-wise error correction through Monte Carlo simulations was adopted, a minimum cluster of 140 voxels for a voxel-level significance of 0.05 led to a surviving cluster at the crosshair (Voxel 1) for XUV for ESM and XUV for ASM. For the cluster labeled with blue circles (Voxel 2), the surviving tests were AUC for ESM, AUC and β_*0*_ for ASM. **(B)** The power differences (*p*-values in blue when below 0.05) among the five tests are demonstrated at Voxels 1 and 2, whose approximate locations (left postcentral gyrus and left precuneus) are marked with the green crosshair and blue circle respectively in the axial views in **(A)**. **(C)** The estimated HDRs through ESM are shown for the two conditions (first two columns) and their differences (third column) at Voxels 1 and 2. Each HDR profile spans over 11 TRs or 13.75 s. The profile patterns at Voxels 1 and 2 are shared by their neighboring voxels in their respective clusters. In addition to the statistical significance in **(A)** and **(B)**, the HDR signature profiles provide an extra evidence for the associated effects at these voxels.

How do the testing approaches perform when applied to real data? Would their performances be consistent with the simulations? To address these questions, we ran 3dMVM on the ESM data presented in the Introduction section with *n* = 50 (2 groups: 21 children and 29 adults), *m* = 20 (2 conditions with each having 10 component estimates at 10 TR grids) and design matrix ***X*** of *q* = 4 columns in the MVM (1): all ones (intercept associated with the average effect across groups), effect coding for the two groups, the average age effect between the two groups, and the interaction group:age (or group difference in age effect). The age values were centered within each group so that the group effect can be interpreted as the difference between the two groups at their respective average age. The effect of interest was on the interaction of group and condition: Did the two groups have the same HDR profile difference between the two conditions? Five F-statistics from MVT, XUV (with sphericity correction), AUC, L2D, and XMV, were obtained and then, due to different degrees of freedom, converted to Z-values for direct comparisons (Figure 2A). To take advantage of the geometrical representation in Table 1 when interpreting the effect of interest, we reduce the within-subject factor Condition to the contrast between the two conditions, so that the interaction effect essentially becomes the group contrast in terms of the HDR profile difference between the two conditions (Figure 2C).

Consistent with the simulation results, XUV achieved the highest detection power in most regions (Figure 2A top) while L2D showed low power (and likely high FPR) due to no differentiation between the positive and negative effect estimates for ESM. All the other three methods, MVT, AUC, and XMV, were generally less powerful than XUV. The strong performance of XUV can be seen in the estimated HDR curves at Voxel 1 (Figures 2B left,C) extracted from a cluster (left postcentral gyrus). More specifically, the adults had roughly the same HDR profile between the two conditions except for a faster recovery phase under the Congruent condition than the Incongruent condition; in contrast, the upstroke and peak were more elevated under the Congruent condition in the children than the Incongruent condition except for the recovery phase during the last 3 TRs. Geometrically, the interaction effect between Group and Condition at Voxel 1 is represented by the fact that the HDR profiles of condition difference were intersecting between the two groups (Figure 2C). MVT and XMV achieved a moderate power while AUC and L2D failed to reach the significance level of 0.05 at Voxel 1 (Figure 2B left). On the other hand, the detection failure of XUV at Voxel 2 (left precuneus) was caused by the fact that the condition contrast was roughly parallel between the two groups (Figure 2C), as geometrically demonstrated in Table 1. MVT, AUC, and XMV showed their auxiliary role when XUV failed (Figure 2B left).

With the ASM analysis results, five tests were performed using 3dMVM. First, the popular approach of focusing on the effect estimate β_0_ associated with the first basis (canonical) function through the hypothesis (6b) was adopted (Figure 2A bottom). Secondly, the L2D approach (7) was used on the first two basis functions (not shown here) as well as all three. Thirdly, MVT was performed using (2b) with the three coefficients. Lastly, the HDR curve at each condition was reassembled for each subject using the three coefficients, and the reconstructed effect estimates only at the first 10 TRs were analyzed with 3dMVM for two reasons: a) with the three SPM curves covering 32 s or 25 TRs, the model would contain too many parameters relative to the data size; b) the effect estimates after the first 10 TRs were mostly negligible. Two tests, XUV and AUC, were performed while MVT and XMV were impossible because the rank was 3 among the 10 effect estimates from the linearly reconstructed HDR per condition.

The detection power for both β_0_ and L2D with ASM was very low (Figure 2A bottom), illustrating the fact that focusing on the peak or the combined effects associated the two or three basis functions would largely fail to detect subtle differences during the BOLD uprising and recovery phases. In contrast, MVT (with the coefficients from three basis functions of ASM), XUV and AUC (with the reconstructed HDRs from ASM) outperformed the conventional approaches of β_0_ and L2D in SPM. Such failure of ASM is specifically demonstrated at Voxel 1 where the peak alone or the summarized values from the three coefficients were not as powerful as the reassembled HDR profiles (Figure 2B right). It is noteworthy that XUV with ASM was less powerful than its ESM counterpart, showcasing the coarser characterization with three parameters in ASM than the estimation at every time point in ESM. Furthermore, for both ESM and ASM, even though XUV was mostly more powerful than the alternatives, MVT and AUC (as well as XMV for ESM and β_0_ for ASM) played a supplementary role when XUV failed (Voxel 2 in Figure 2B right).

To recapitulate the performance of the five testing methods in situations when LME cannot be applied, ESM provided a more accurate estimation for the HDR curves than ASM, leading to a higher success in detection power. In addition, with the typical sample size in most studies, XUV as an approximate approach had the lowest power loss at the group level compared to other dimensional alternatives as well as the test with the most accurate hypothesis formulation, MVT. However, MVT plus the lesser accurate approximations such as AUC and XMV may play an auxiliary or even irreplaceable role in situations when XUV suffers from power loss (e.g., Table 1 or Voxel 2 in Figure 2).

## Discussion

There are many characteristics that could describe the HDR shape: onset latency, onset-to-peak, peak location, peak duration, magnitude or shape of the undershoot after the onset or during the recovery phase, and habituation or saturation effect. Because of the multiple facets of HDR shape, a lot of effects may well have gone undetected at both individual and group levels in most neuroimaging data analyses, and the failures to capture the shape nuances might have partially contributed to the poor reliability and reproducibility in the field. With a few exceptions, most analyses adopt FSM or ASM mainly for the simplicity of group analysis, as each condition or task is associated with one effect estimate, while other coefficients (e.g., time and dispersion derivatives in ASM) are a priori ignored. That is, activation detection intuitively focuses on the estimated magnitude around the activation peak while statistical inference on the whole HDR shape is generally considered a daunting hurdle. FSM may work well for situations such as a contrast between a condition and fixation. However, it would fail to detect shape subtleties such as prolonged plateau at the peak, slower or faster rise or fall, bigger or longer undershoot, or overall duration. Therefore, FSM through a presumed HDR (gamma variate in AFNI, canonical function in FSL and SPM) is very crude even in an experiment with a block design (Saad et al., 2006; Shan et al., 2013). ASM is an improvement over FSM; however, its flexibility is still limited. For instance, when one is interested in contrasting two conditions (or groups) or in investigating higher-order interactions, the three ASM basis functions may still not be enough in capturing the undershoot subtleties. In addition, characterizing the whole HDR curve with its peak value from ASM for group analysis may suffer from significant power loss, as demonstrated in our real experimental data. Response shapes can vary considerably over space (e.g., Handwerker et al., 2004; Gonzalez-Castillo et al., 2012; Badillo et al., 2013), and we believe it is important to model more accurately the HDRs at the individual level and test for shape rather just amplitude at the group level, particularly when detecting subtle differences between conditions or groups. The dominant adoption of FSM or ASM with a relatively rigid HDR shape reflects the daunting challenge in adopting ESM at the group level, and it is this challenge that motivated our exploration of various group analysis strategies with ESM.

### Overview of the testing methodologies

Among all the testing strategies for ESM (Table 1), MVT and LME maintain an accurate characterization for the hypothesis. In contrast, the dimensional reduction methods AUC, L2D, and EXC (XUV and XMV) project the original space of the alternative hypothesis from ℝ^*m*^ to ℝ^1^, ℝ^1^, and ℝ^*m*−1^, respectively. Any dimensional reduction usually translates to information loss or geometrical distortion. Based on the results from our simulations and real data applications, we believe that the major testing methods for ESM are LME, XUV, MVT, XMV, and AUC, which all have the proper controllability for FPR. If sample size is not an issue in FMRI studies, MVT (e.g., hypothesis 2a or 2b) would be the most accurate approach in terms of hypothesis characterization. However, in practice the number of subjects is usually not large enough for MVT due to resource limitations (e.g., financial cost, time, and manpower), leading to an underpowered performance of MVT as shown in our simulations and real data. Among all the workaround methods through dimensional reduction, XUV has the least hypothesis distortion and the lowest power loss. With one group of subjects and no other explanatory variables present, XUV surpasses MVT, XMV, and AUC in power. However, with an accurate representation of the hypothesis, LME is slightly more efficient than XUV, and should be considered as the first choice (e.g., Alvarez et al., 2008). For all other situations, LME modeling is not feasible due to the constraint of variable parameterization, and we opt for the workaround methods through dimensional reduction, among which AUC is insensitive to subtle shape differences while XMV mostly underperforms unless when the temporal correlation is relatively high (e.g., 0.65 or higher; Chen et al., 2014). XUV achieves the best balance between dimensional reduction and statistical power. However, as XUV tests for parallelism, not exactly the same as the accurate representation characterized in MVT, it may fail in detecting the situation where the HDR profiles are roughly parallel. To compensate for the occasions when XUV fails, other dimensional reduction methods (MVT, AUC, XMV) may offer some complementary detection power.

In light of the discussion here, we strongly encourage the adoption of the ESM approach to achieving two goals: detecting activations and estimating the hemodynamics by characterizing the HDR shape. In addition to the large power gain at both individual and group levels, ESM provides the estimated HDR shape information at the group level, providing an extra layer of validation about the effect veracity through the graphical display of the familiar HDR shape, and alleviating the misconceptions and malpractices prevalent in statistical analysis (e.g., P-hacking, graphical presentation of statistic values instead of effect estimates, overuse of statistical significance; Motulsky, 2014). The HDR profile information from ESM offers a precious boost especially when a cluster fails to survive the typical stringent thresholding for multiple testing correction but still reaches the significance level of 0.05 at the voxel level. Such a reassuring support of ESM is unavailable from the alternatives of FSM and ASM, with which typically the investigator would be only able to report the peak HDR magnitude or statistic values at a region.

Our recommendation of adopting ESM not only applies to event-related experiments, but also are adaptable to modeling the attenuation or habituation effect in block designs (Saad et al., 2006). In addition, this approximation modeling methodology of XUV assisted with MVT, AUC, and XMV has been applied to DTI data in which the simultaneous variables (white matter network groups such as corpus callosum, corona radiata, left and right hemispheric projection fibers, left and right hemispheric association fibers) were modeled by multiple explanatory variables (e.g., sex, age, behavioral measures) for each response variable such as fractional anisotropy, axial diffusivity, mean diffusivity, radial diffusivity, T1 relaxation time, proton density, and volume (Taylor et al., 2015).

The proposed modeling strategies have been implemented into the open-source program 3dMVM in AFNI, which offers the investigator all the testing results in the output including XUV and the auxiliary approaches (MVT, XMV, and AUC). MVT for the components from ESM presents a unique challenge when one or more within-subject factors are included in the model, and we offer a testing strategy that still fits in the MVM framework (Appendix B). As an alternative, these tests could be conducted in the traditional univariate GLM except for the two multivariate methods, MVT and XMV. In other words, some of the testing methods (MVT and XMV) are truly multivariate, while others (XUV, AUV, and L2D) are essentially univariate. However, as we demonstrated in Chen et al. (2014), these univariate tests are sometimes difficult to perform under the univariate framework, as shown by the implementation challenges faced by some of the neuroimaging packages. Instead, these univariate tests can be more conveniently formulated under the MVM platform by treating the levels of each within subject factor as simultaneous variables in (1) and then constructing the univariate testing statistics through a conversion process. For example, those univariate tests presented in Figure 2 cannot be performed under the univariate GLM framework due to the incorporation of a covariate (age) in the presence of two within subject factors (Condition and HDR effects). It is in this sense that we frame our discussion here under the MVM perspective.

### Limitations of the ESM approach

It is noteworthy that the reliability information from the individual subject analysis is not considered at the group level with the modeling methods discussed here, unlike the mixed-effect multilevel analysis (Worsley et al., 2002; Woolrich et al., 2004; Chen et al., 2012). In addition, the number of basis functions monotonically increases among FSM, ASM, and ESM, therefore it is expected that the goodness of fit at the individual subject analysis level improves across the three methods. On the other hand, as each condition is characterized through multiple (e.g., ≥7) basis functions in ESM, a reliable estimation of the HDR curve at the individual level pays a price through the lower degrees of freedom and requires enough (e.g., 20 or more) trials per condition, and may encounter the risk of numerical instability due to high correlations or even multicollinearity among the regressors. These latter issues can be exacerbated by poor stimulus timing designs. In addition, the typical regression analysis at the individual level assumes the linearity of HDR across trials. Although available (e.g., 3dNLfim in AFNI), a non-linear approach is usually difficult to handle and still requires some extent of prior information about the HDR shape. Furthermore, the ESM approach is generally considered to be susceptible to noise or effects unrelated to the effects of interest (e.g., head motion, physiological confounds). In other words, the confounding effects may leak into the HDR estimation through over-fitting. However, the false positives from the potential over-fitting at the individual level is less a concern at the group level for the following reasons: a) the likelihood is reduced unless most subjects systematically have similar or same confounding effects; b) cluster-based inferences may reduce the risk of false positives; and most importantly c) examination of the estimated HDR profiles offer an extra safeguard to filter out the potential false positives.

### Comparisons with other modeling approaches

Some (not all) of the dimensional reduction methods for ESM discussed here have been sporadically and individually applied to real data in the literature. For example, a popular practice with ASM is to solely focus on the coefficient of the principal basis function (e.g., canonical curve in SPM) with other coefficients (e.g., time and dispersion derivatives) being a priori abandoned. As our results with real data showed, the investigator may fail to detect most activations when the effect lies in the HDR shape nuances but not the peak. One suggestion for ASM was to extend the definition of amplitude in (6) to the *L*^2^-distance by including either the effect for the time derivative (7) or the effects for both time and dispersion derivatives (8) (Calhoun et al., 2004; Worsley and Taylor, 2006; Steffener et al., 2010). A similar approach was to express the effect estimates from the first two basis functions of ASM as a complex number (Wang et al., 2012). However, the potential issues with L2D or its analogs (e.g., Worsley and Taylor, 2006) are the following. a) The definition of amplitude extension in (7) and (8) is under the premise that all the three basis functions are orthogonal with each other (Calhoun et al., 2004). However, only the first two basis functions are orthogonal with each other, but not the third one. b) The second and third basis functions are not normalized; that is, they are not scaled to have a maximum value of 1, unlike the first basis function. In addition, the three effect estimates have different dimensions: the first is of percent signal change while the other two of percent signal change by the unit of time. Therefore, it is difficult to render a physically meaning interpretation with the L2D measures. c) All the effect estimates including negative values are folded into a positive L2D measure, which cannot be differentiated among those effect estimates on the same circle or sphere (see Table 1). In addition, it may lead to the violation of the Gaussian distribution assumption, as illustrated in the poor controllability of FPR (Figure 1). d) Their power performance is not satisfactory (Figures 1, 2). As an alternative, MVT or LME through the hypothesis (2a) or (2b) on the two or three effect estimates from ASM, as shown in Figure 2A, provides a more accurate characterization because it allows for different units or dimensions across the effects.

Similarly for ESM, two dimensional reduction methods have separately been adopted in data analyses. For example, AUC was employed in Beauchamp et al. (2003), Greene et al. (2007), and McGregor et al. (2013). Although not explicitly stated, XUV was used in several real applications to identify the HDR effect under a condition through the main effect (or one-way interaction) of the ESM components in a one-way within-subject ANOVA (Weissman et al., 2006; Geier et al., 2007; Church et al., 2008), to detect the group or condition differences in the overall HDR shape through the group-by-component or condition-by-component interaction in a two-way ANOVA (e.g., Schlaggar et al., 2002; Church et al., 2008; Shuster et al., 2014), and to explore the three-way group-by-task-by-component interaction (Church et al., 2008). However, two limitations were not addressed in those analyses: the potential identification failure of XUV (Table 1 and Voxel 2 in Figure 2), and the limited applicability of univariate GLM.

Some comparisons were performed in terms of amplitude, peak latency, and duration in the estimated HDR among various modeling methods (e.g., FSM, L2D, ESM, a nonlinear model, and inverse logit model; Lindquist et al., 2009). The inverse logit model was deemed the best among the candidates in both simulations and real data, and slightly more powerful than ESM. However, the comparisons were not optimal. First, the dimensional reduction from the HDR shape in ℝ^*m*^ to the three quantities (amplitude, delay, and duration) in ℝ^3^ might be compromised in power when detecting the shape subtleties—this point can be highly dependent on the experiment. Secondly, the reliability for the estimation of the three characteristics was suboptimal. For example, the lackluster performance of ESM in Lindquist et al. (2009) might be caused by the inaccurate amplitude based on the first local peak because such an approach could be misleading especially when more than one local peak occurs. Lastly, the final group analyses were still focused on the amplitude with the Student's *t*-test, an effective dimensional reduction from ℝ^*m*^ to ℝ^1^.

A multivariate approach (Zhang et al., 2012) was previously proposed, analogous to our method except for the following differences. It was demonstrated among the voxels within only five structurally pre-defined regions; smoothing the estimated HDR from each subject by a Gaussian kernel and imposing regularization on the smoothed HDR were performed to improve the temporal continuities of the HDR; and group analysis was run through multivariate testing of one-sample or pair-wise comparisons among conditions, equivalent to MVT (2a or 2b) discussed here. Another approach (Zhang et al., 2013) assumed that the HDR under each condition would only vary in amplitude and latency across subjects; that is, the HDR shape was presumed same across all subjects. Specifically, the HDR curve for each condition was characterized at the group level by two parameters: one was of interest (amplitude) and the other of no interest (delay). In addition, the HDR shape (fixed across subjects) was modeled by cubic splines plus their time derivatives. Once the amplitude was estimated for each subject in a one-tier model that incorporated both within- and across-subject variances, a second round of group analysis was performed only on the amplitudes (ignoring the delay) through typical one-sample or paired *t*-test to make inference about a condition or contrast. The approach was demonstrated among the voxels within only three structurally predefined regions.

Recently, a hierarchical approach was proposed for ESM through integrating both individual and group levels into one model (Degras and Lindquist, 2014) in which the HDR curves were captured through multiple higher-order B-spline functions. Even though only demonstrated on one slice of data, the approach is appealing because the variability at both levels is accounted for. However, the current implementation in Matlab is hindered by the following constraints or limitations. a) Spatial parcellation based on anatomical structure was required to determine the temporal correlation structure in the noise component. More applicable approaches would be based on a priori regions that are functionally parcellated through, for example, hierarchical clustering (Thirion et al., 2006; Ji, 2010), joint parcellation detection-estimation (Badillo et al., 2014), consensus clustering (Badillo et al., 2013), k-means clustering (Ji, 2010), etc. (b) The HDR shape may vary across different stimulus conditions under some scenarios (e.g., Ciuciu et al., 2003), and a presumption of the same shape HDR as in Degras and Lindquist (2014) may decrease the detection power when the shape subtleties are of interest. The same HDR assumption is reasonable under other circumstances and has proven sufficient for encoding or decoding the brain activity (Pedregosa et al., 2015). c) Final statistical inference in Degras and Lindquist (2014) through an asymptotic *t*-test was still based on the scaling factors of the same HDR curve shared by all conditions, a dimensional reduction approach from ℝ^*m*^ to ℝ^1^. An alternative approach is the incorporation of both individual and group levels in a mixed-effects model under the Bayesian framework (Chaari et al., 2013; Badillo et al., 2014). Applied at a priori regions that are functionally parcellated, this jointed detection and estimation method may render a robust procedure less sensitive to outliers than the conventional two-tier methods under the assumption that all the voxels share the same HDR within a region or parcel.

## Conclusion

Here we demonstrate with simulations and experimental data that the fixed-shape (FSM) or adjusted-shape (ASM) method may fail to detect most of the shape subtleties (e.g., the speed of rise or recovery, undershoot) in hemodynamic response (HDR). In contrast, the estimated-shape method (ESM) through multiple basis functions would more accurately characterize the cerebral blood flow regulation, and significantly improve the detection power at both individual and group levels. In addition, we propose an analysis scheme for ESM that still fits within the conventional two-tier analysis pipeline and achieves higher statistical power than the alternatives: one performs regression time series analysis separately for each individual subject, and then conducts group analysis with the individual effect estimates. For one group of subjects, a linear mixed-effects (LME) model is preferred if no other explanatory variables are present. In all other scenarios, statistical inferences on the HDR shape can be achieved through a hybrid combination of multivariate testing (MVT) and dimensional reduction approaches with a multivariate model (MVM). Simulations are shown in terms of controllability for false positive rate (FPR) and power achievement among various testing methods. The strategy was applied to a dataset from a real experiment to compare among different testing strategies in terms of power assessment. In addition, we showcase that the MVM flexibility allows any number of explanatory variables including between- and within-subject factors as well as between-subjects covariates.

### Conflict of interest statement

The authors declare that the research was conducted in the absence of any commercial or financial relationships that could be construed as a potential conflict of interest.

## Footnotes

^1^The efficiency in the statistics context measures the optimality of a testing method. A more efficient test requires a smaller sample size to attain a fixed power level.

## Appendix A

### List of acronyms used in the paper

**Table:**

AN(C)OVA	Analysis of (co)variance
ASM	Adjusted-shape method
AUC	Are under the curve
ESM	Estimated-shape method
EXC	Effect-by-component interaction
FPR	False positive rate
FSM	Fixed-shape method
GLM	General linear model
HDR	Hemodynamic response
IRF	Impulse response function
L2D	Euclidian (*L*^2^) distance
LME	Linear mixed-effects
MAN(C)OVA	Multivariate analysis of (co)variance
MVM	Multivariate modeling
MVT	Multivariate testing
UVM	Univariate modeling
UVT	Univariate testing
XMV	Multivariate testing for interaction
XUV	Univariate testing for interaction.

## Appendix B

### Formulation of multivariate testing in the presence of one or more within-subject factors

As discussed in Chen et al. (2014), all the within-subject factors are flattened into ℝ^1^ under the multivariate model (MVM) formulation (1). Once the regression coefficient matrix ***A*** is estimated through solving the MVM system (1) with the least squares principle, each general linear test (GLT) can be expressed as a function of ***A***,

(A1) $$H_{0}:L_{u\times q}\;A_{q\times m}\;R_{m\times v}=0_{u\times v},$$

where the *hypothesis matrix **L***, through premultiplying, specifies the weights among the rows of ***A*** that are associated with the between-subjects variables (groups or subject-specific quantitative covariates), and the *response transformation matrix **R***, through postmultiplying, formulates the weighting among the columns of ***A*** that correspond to the *m* response variables. It is assumed that ***L*** and ***R*** are full of row- and column-rank respectively, and *u* ≤ *q*, *v* ≤ *m*. The matrix ***L*** (or ***R***) plays a role of contrasting or weighted averaging among the groups of a between-subjects factor (or the levels of a within-subject factor).

The conventional multivariate test (MVT) can be performed through any of the four multivariate statistics (Wilks' λ, Pillai-Bartlett trace, Lawley-Hotelling trace, and Roy's largest root) with ***R*** = ***I***_*m*_ once the hypothesis matrix ***L*** in (A1) is constructed (Appendix B in Chen et al., 2014). For instance, suppose that we consider an *m*-variate model with the following explanatory variables: three genotypes of subjects, age and their interactions. Via effect coding with the first genotype as reference, the model matrix ***X*** in (1) is of *q* = 6 columns: one for the intercept, two for the three genotypes, one for age, and two for their interactions. Accordingly, the *q* = 6 rows in ***A*** represent the overall mean, the respective effects for the second and third genotypes relative to the overall mean, the age effect associated with the overall mean, and the respective age effects for the second and third genotypes relative the average age effect. MVT for the main effect of genotypes, the genotype-by-age interaction, and the age effect for the first genotype can be obtained under (A1) respectively with

$$\begin{array}{l} L_{1}=\left[\begin{array}{c} \begin{array}{l} 0\;1\;0\;0\;0\;0\\ 0\;0\;1\;0\;0\;0 \end{array} \end{array}\right],L_{2}=\left[\begin{array}{c} \begin{array}{l} 0\;0\;0\;01\;0\\ 0\;0\;0\;0\;0\;1 \end{array} \end{array}\right],\\ L_{3}=\left[\begin{array}{c} 0\;0\;0\;1\;-1\;-1 \end{array}\right],R_{1}=R_{2}=R_{3}=I_{m}. \end{array}$$

Similarly, both univariate and within-subject multivariate tests can be formulated by obtaining both the hypothesis matrix ***L*** and the response transformation matrix ***R*** in (A1) (Appendix C in Chen et al., 2014). In addition, all the *post-hoc t*- and *F*-tests (options -gltCode and -glfCode respectively in 3dMVM) are also constructed as MVT under the platform (A1). For instance, the effect under a specific level and the contrast between two levels of a within-subject factor through -gltCode are evaluated essentially by a one-sample and a paired *t*-test respectively, while the main effect of a within-subject factor through -glfCode is assessed by a within-subject multivariate test.

When ***R*** = ***1***_*m*×1_, the hypothesis (A1) solely focuses on the between-subjects explanatory variables (columns in the model matrix ***X*** of MVM; 1) while the effects among the levels of the within-subject factors are averaged (or collapsed). Therefore, the AUC approach (4) can be conceptually tested under the multivariate framework (A1), respectively for one group,

$$L_{4}=1,R_{4}=1_{m\times 1},$$

and two groups,

$$L_{5}=(0,1),R_{5}=1_{m\times 1},$$

even though they would be readily performed through the conventional one- and two-sample *t*-tests.

When applied to the effect-by-component interaction (9a or 9b) with ESM (EXC in Table 1), the MVM framework offers both univariate (XUV) and multivariate (XMV) approaches, which are tested under the same formulation, respectively for one group (A1),

$$\begin{array}{l} H_{0}:\alpha_{1}=\alpha_{2}=...=\alpha_{m},\\ L_{6}=1,R_{6}=\left[\begin{array}{c} I_{m-1}\\ -1_{1\times (m-1)} \end{array}\right], \end{array}$$

xma and two groups,

$$\begin{array}{l} H_{0}:\alpha_{11}-\alpha_{21}=\alpha_{12}-\alpha_{22}=...=\alpha_{1m}-\alpha_{2m},\\ L_{7}=(0,1),R_{7}=R_{6}. \end{array}$$

For XMV, standard multivariate testing statistics (Wilks' λ, Pillai-Bartlett trace, Lawley-Hotelling trace, Roy's largest root) are constructed through the eigenvalues of the “ratio” ***H***(***H*** + ***E***)^−1^ between the SSPH matrix ***H*** for the hypothesis (A1) against the SSPE matrix ***E*** for the errors in the full model (Rencher and Christensen, 2012). In contrast, the univariate approach XUV is tested through the formulation of an *F*-statistic with the numerator and denominator sums of squares being as *tr*(***H***(***R***^*T*^ ***R***)^−1^) and *tr*(***E***(***R***^*T*^***R***)^−1^) under the sphericity assumption (Fox et al., 2013), and the *F*-value can be adjusted through the Greenhouse and Geisser (1959) or Huynh and Feldt (1976) correction if the sphericity assumption is violated.

All the applications so far in the literature have been focused on either MVT or UVT. In other words, a strict MVT applies to the situations of truly multivariate nature while a purely UVT is adopted to the conventional AN(C)OVA or GLM. However, if we treat the components from ESM as simultaneous response variables, the presence of one or more within-subject factors (e.g., two task conditions in the experimental data of this paper) necessitates a partial MVT. Here we demonstrate a strategy to formulate partial MVT with the construction of ***L*** and ***R*** using a template of two-way within-subject ANOVA with factors *A* and *B* of *a* and *b* levels respectively. Suppose that we want to model the levels of factor *A* as *a* simultaneous response variables (e.g., components or effect estimates from ESM) while factor *B* is considered as an explanatory variable (e.g., conditions). MVT for the effect of *B* can be achieved through the following specifications in (A1),

$$L=I_{q},R=I_{a}⊗R^{\left(B\right)}.$$

Similarly, if the levels of factor *B* are modeled as *b* simultaneous response variables while factor *A* is considered as an explanatory variable, we have the following MVT specifications for the effect of *A*,

$$L=I_{q},R=R^{\left(A\right)}⊗I_{b}.$$

The notations $R^{\left(A\right)}=\left[\begin{array}{c} I_{a-1}\\ -1_{1\times (a-1)} \end{array}\right]$ and $R^{\left(B\right)}=\left[\begin{array}{c} I_{b-1}\\ -1_{1\times (b-1)} \end{array}\right]$ above are conveniently the effect coding matrices for factors *A* and *B* respectively.
